# A Plumb Fit

**DOI:** 10.5811/cpcem.2017.9.35222

**Published:** 2017-10-18

**Authors:** Michael F. Harrison, Kevin Rooney, Bradley Jaskulka

**Affiliations:** * Henry Ford Hospital, Department of Emergency Medicine, Detroit, Michigan; † Henry Ford Hospital, Department of Internal Medicine, Detroit, Michigan; ‡ Henry Ford Hospital, Department of Internal Medicine, Division of Critical Care Medicine, Detroit, Michigan

## CASE PRESENTATION

In the middle of the night, a young male with no significant past medical history presented from a local detention facility with an unusual chief complaint – entrapment of his right hand in the cell’s stainless steel toilet basin. The circumstances leading to this complaint were unclear, and the hand had been in the toilet for approximately three hours at the time of arrival. When detention facility staff, including a plumber, were unsuccessful in freeing the hand, the patient and the entire toilet and sink assembly were transported to our emergency department (Image). To assist efforts to safely remove the hand, a plain radiograph identified its location with respect to the toilet’s inner structure (Image).

While preparations were being made to cut the toilet with a power saw, approximately 500mL of ultrasound gel was applied to the basin and allowed to seep into the outflow tract. Using firm manual traction, the patient’s hand was then safely freed. Physical exam of the liberated hand revealed water aging but no other anatomical, functional, or sensory abnormalities. A subsequent (more traditional) series of plain radiographs revealed no acute osseous injury; and laboratory analysis, including creatinine phosphokinase levels, were within normal limits. The patient and intact toilet were subsequently discharged to the detention center.

## DISCUSSION

While using a toilet may seem like a benign common process, injuries do occur.^1^–^3^ Alternatively, hand injuries are one of the most common complaints of prisoners requiring medical attention.^4^–^6^ Regardless of patient population, this case demonstrates an unusual marriage of hand and toilet injuries requiring medical intervention. The use of ultrasound gel to liberate the patient’s hand is an excellent example of the “thinking on your feet” skillset that makes our profession both challenging and enjoyable.

**CPC-EM Capsule**

What do we already know about this clinical entity?

As emergency physicians, we know people often get their hands or other appendages entrapped in usual manners and places. They come to us for help.

What is the major impact of the image(s)?

The current image demonstrates an unusual manner of manual entrapment with an unorthodox use of lubrication and radiographs. We can provide solutions.

How might this improve emergency medicine practice?

The ability to maintain professional composure while thinking on our feet and outside the box defines a successful emergency physician. These cases also make the job fun.

## Figures and Tables

**Image:**
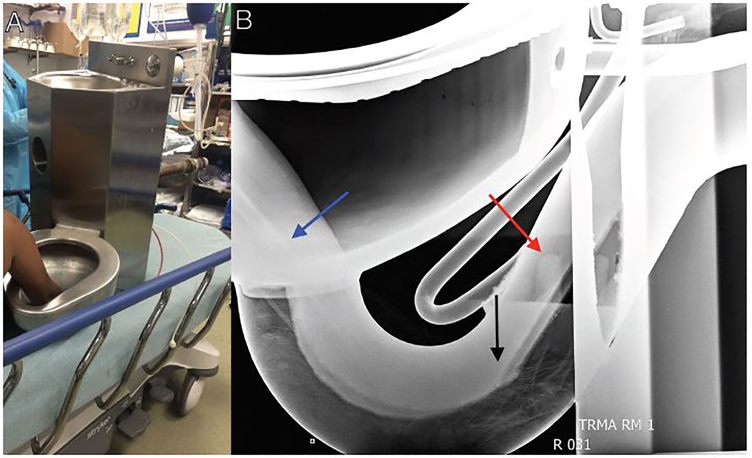
The patient’s right hand and intact sink/toilet assembly (Panel A) on the stretcher in the trauma bay; the lateral view radiograph of the entrapped hand (blue arrow – shaft of radius; black arrow – metacarpals; red arrow – distal tips of fingers) inside the toilet (Panel B).
